# Supplementary figures and images for: A detailed analysis of game statistics of professional tennis players: An inferential and machine learning approach
Source: PLoS One. 2024 Nov 5;19(11):e0309085. doi: 10.1371/journal.pone.0309085 (PMC11537396; doi:10.1371/journal.pone.0309085)

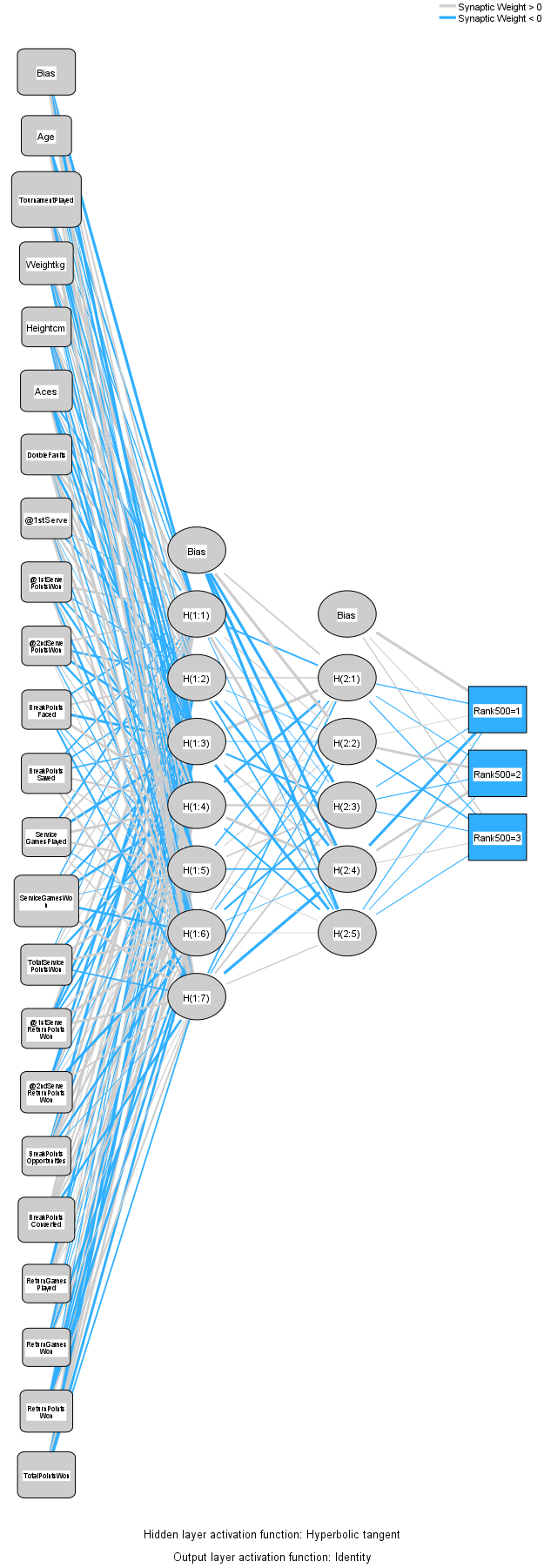

Supplement: S1 Fig — (TIF) [file pone.0309085.s001.tif]
