# Supplementary material for: A detailed analysis of game statistics of professional tennis players: An inferential and machine learning approach
Source: PLoS One. 2024 Nov 5;19(11):e0309085. doi: 10.1371/journal.pone.0309085 (PMC11537396; doi:10.1371/journal.pone.0309085)
Supplement: Attachment — Submitted filename: Response to Reviewers.docx [file pone.0309085.s002.docx]

**Journal Requirements**

PLOS ONE's style requirements, including those for file naming.

We corrected the manuscript according to the required styles.

Please note that PLOS ONE has specific guidelines on code sharing for submissions in which author-generated code underpins the findings in the manuscript. In these cases, all author-generated code must be made available without restrictions upon publication of the work. Please review our guidelines at <https://journals.plos.org/plosone/s/materials-and-software-sharing#loc-sharing-code>

and ensure that your code is shared in a way that follows best practice and facilitates reproducibility and reuse.

The code for the best-generated model is publicly available on the Figshare repository and is also cited in the text and references of the manuscript; Anonymized. NN 3.1 model. Figshare. 2023. doi:10.6084/m9.figshare.24762705.v1. Upon the completion of the review process, the term 'Anonymized' will be replaced with the authors' names. Since the code file is under the CC BY 4.0 license (and was uploaded at the end of 2023), it has had 46 views as of today (18 June 2024), which supports its formal availability.

Thank you for uploading your study's underlying data set. Unfortunately, the repository you have noted in your Data Availability statement does not qualify as an acceptable data repository according to PLOS's standards.

At this time, please upload the minimal data set necessary to replicate your study's findings to a stable, public repository (such as figshare or Dryad) and provide us with the relevant URLs, DOIs, or accession numbers that may be used to access these data. For a list of recommended repositories and additional information on PLOS standards for data deposition, please see <https://journals.plos.org/plosone/s/recommended-repositories>.

The data necessary for replicability of the results are available on the public repository recommended by PLOS ONE, Figshare. The link is provided in the text (Data Accessibility section) and in the references. Currently, the authors' names are replaced with 'Anonymized', which will be updated after the completion of the review process. Since the data are under the CC BY 4.0 license (and were uploaded at the end of 2023), they have had 83 views as of today (18 June 2024), which supports their formal availability.

The main issue has been noticed by the reviewers is the writing problem. The manuscript has not been organised well and the contents are not clear. The motivation of the research work is not described well and the results lack of analysis. Thus, a proof reading is needed to clarify the concerns from the reviewers and the innovation should be highlighted clearly in the revised version

Upon thorough rereading of the manuscript and after overcoming authorial blindness, we agree with this assertion from the editor and reviewers. We have corrected the manuscript accordingly. Thank you for bringing this significant issue to our attention.

**Reviewer #1**:

Figure 7 Writing is not clear.

Thank you for your comment. We agree that the text in Figure 7 was illegible, and therefore, we have decided to split the figure into S1 Fig (NN diagram, panel a) and Figure 7, containing only the ROC Curve for the Best Performing Model 3.1 (panel b). This adjustment ensures that the crucial data for the credibility of the data analysis process are clearly presented.

Language article clear, correct.

Thank you

The article is in pdf version.

As we uploaded the manuscript as a doc. (MS Word) file, we believe it was automatically converted to PDF by the PLOS One software. Unfortunately, we are unable to address this issue.

If the word would be better for me to confirm some things, it should be more justified.

We justified some text at the request of other reviewers. We hope that the text will now be more readable and that his claims will be confirmed. Thank you for your feedback.

In all tables follow the same guidelines for formatting.

Thanks for your feedback, we have unified the formatting and adjusted the style of the notes according to the PLOS ONE guidelines that were also brought to our attention by the editor.

**Reviewer #2:**

The abstract needs to be specific on how the data was collected with the design of the study

Thank you for pointing out this shortcoming, which occurred because we tried to reduce the number of words in the abstract. Based on your valid comment, we have added information to the abstract about the selected research design (retrospective predictive study) and the fact that it was secondary data and the method of verification of erroneous values.

The introduction needs to be redone. Line 43 is not clear. what does these mean? The introduction has all mixed information which does not clearly show what the authors are focusing at. the aim of research is not clear.

When we re-read the contents of the Introduction after some time. We have to admit that the information we provided in it was confusing and, in some cases, unnecessary for the current research plan and study objectives. Thanks for pointing this out. We deleted unnecessary information from the manuscript (for example, in the first paragraph, where we characterize tennis using terms that we no longer work with). We have continuously incorporated the context and links to the current goals of the study into the text.

Furthermore, we simplified and refined the formulation of the research aim of this study. The previous version focused too much on AI, which was only supposed to be a tool to answer research questions. We thank the reviewers for pointing out this deficiency.

**Reviewer #3:**

I respect the effort been made from the author. The abstract introduces the significance of using Artificial Intelligence, specifically Neural Networks to help ATP ranking with using all factors of ATP's ranking, instead of going through long process.

Thank you for your review. We have incorporated your note into the manuscript; "instead of going through a long process". As this precisely characterizes the purpose of our study.

I can see this paper was written well, but the author need to add more information in (Statistical analysis section) in Utilizing the Multivariate Analysis of Variance.

We add more detailed characteristic of MANOVA steps and the procedures we performed. We hope that this way it will be clearer, more replicable and the reader will get more detailed information about the process of data analysis using the MANOVA test. Furthermore, we unified the naming of Partial eta squared (from η² to η²p).

Also, the section (Neural Network Analysis of Multivariate Outcomes Associated with ATP Rank) lacks some details, and need to be more clear.

Thank you for your feedback. We cleaned up the content and reformulated selected parts. Unfortunately, since it is largely a description of the results, but mainly of the process, which for ethical reasons and for the sake of reproducibility we must mention, these changes were not as extensive as, for example, in the case of the introduction, research objectives, and Statistical analysis section.

Other than that I can see all other information been added as it should be.

Thank you and the other reviewers and the editor for dedicating your valuable time to our article. We believe that your efforts and comments have indeed enhanced the quality of our study, for which we are immensely grateful.
